# Lipid desaturation-associated endoplasmic reticulum stress regulates *MYCN* gene expression in hepatocellular carcinoma cells

**DOI:** 10.1038/s41419-020-2257-y

**Published:** 2020-01-27

**Authors:** Xian-Yang Qin, Ting Su, Wenkui Yu, Soichi Kojima

**Affiliations:** 1 Liver Cancer Prevention Research Unit, RIKEN Cluster for Pioneering Research, Wako, Saitama 351-0198 Japan; 2 Department of Intensive Care Unit, the Affiliated Drum Tower Hospital, Medical School of Nanjing University, Nanjing, Jiangsu 210008 China

**Keywords:** Cancer metabolism, Gastrointestinal cancer

## Abstract

Hepatocellular carcinoma (HCC) is the second leading cause of cancer-related deaths worldwide due to its high rate of recurrence, in part because of cancer stem cell (CSC)-dependent “field cancerization”. Recently, we identified that the oncogene v-myc avian myelocytomatosis viral oncogene neuroblastoma derived homolog (MYCN) marked CSC-like subpopulations in heterogeneous HCC and served as a therapeutic target and prognostic marker for HCC. In this study, we explored the molecular basis of upregulated *MYCN* gene expression in HCC cells. Liquid chromatograph time-of-flight mass spectrometry-based metabolome analysis demonstrated that the content of unsaturated fatty acids was increased in MYCN high expression (MYCN^high^) CSC-like HCC cells. Inhibition of lipid desaturation using either the chemical inhibitor or siRNA/shRNA against stearoyl-CoA desaturase-1 (SCD1) suppressed cell proliferation as well as *MYCN* gene expression in MYCN^high^ HCC cells, grown as both monolayer and spheres. Further mechanistic study using RNA-seq based transcriptome analysis revealed that endoplasmic reticulum (ER) stress related signaling networks such as endocannabinoid cancer inhibition pathway were under the control of SCD1 in MYCN^high^ HCC cells. Furthermore, the expression of ER stress-inducible transcription suppressor cyclic AMP-dependent transcription factor (*ATF3*) was downregulated in MYCN^high^ CSC-like HCC cells and CSC-rich spheroids, which was upregulated by inhibition of lipid desaturation or treatment with acyclic retinoid (ACR). Lipid profiling using NMR spectroscopy revealed that the ACR dramatically reduced the content of unsaturated fatty acids in HCC cells. The chemical inducer of ER stress inhibited *MYCN* gene expression, while the chemical inhibitor of ER stress or knockdown of *ATF3* gene expression partially rescued the suppression of *MYCN* gene expression by ACR in MYCN^high^ HCC cells. These data suggested that lipid desaturation-mediated ER stress signaling regulates *MYCN* gene expression in HCC cells and serves as a promising therapeutic target for the treatment and prevention of HCC.

## Introduction

Hepatocellular carcinoma (HCC) is a well-characterized inflammation-driven cancer^1^ and is the second most lethal cancer worldwide due to its poor prognosis^2^. Persistent infections of hepatitis B virus (HBV) and hepatitis C virus (HCV) are the major risk factors for HCC^3^. Advances in antiviral therapy have reduced the risk of developing both HBV-^4^ and HCV-related HCC^5^. However, obesity-associated inflammation is responsible for increased death rates for all the cancers^6^. Non-alcoholic steatohepatitis (NASH), which is characterized by continuous hepatocyte death and compensatory proliferation^7^, has attracted much attention and is believed that it will soon be the leading etiology of HCC^8^.

Lipogenesis is known to be high in individuals with NASH and its upregulation is associated with high risk and poor prognosis of NASH-related HCC^9^. Lipid metabolic reprogramming allows the cells to adapt to the tumor microenvironments. For example, lipogenesis may promote the cell growth and proliferation of MYC-driven cancers^10^, probably by maintaining the rebalanced nutrient supply and demand for cellular hyperproliferation^11^. In addition, lipid accumulation in hepatocytes selectively induces lipotoxicity in intrahepatic CD4^+^ T cells and promotes HCC development by evading immune surveillance^12^. Furthermore, there is growing evidence about the role of unsaturated fatty acids in tumor initiation by regulating the generation and maintenance of cancer stem cells (CSCs) or tumor-initiating cells (TICs)^13^.

Tissue regeneration and tumorigenesis share common molecular pathways in the regulation of cell growth and death^14^. In response to resection or injury, healthy liver has the unique ability to grow back or regenerate by a process of compensatory hyperplasia^15^. In contrast, under chronic inflammation such as lipid-rich environment, repeated liver injury and compensatory proliferation might lead to aberrant stabilization and chronic activation of the oncogenes, which can be considered as one of the leading causes of HCC. The MYC family members are critically involved in the regulation of multiple biological processes, including cell growth, proliferation, apoptosis, energy metabolism, and differentiation^16^, and play dual roles in regulating both hepatocellular proliferation and hepatocarcinogenesis^17^. *v-myc* avian myelocytomatosis viral oncogene neuroblastoma derived homolog (*MYCN*), is a well-recognized oncogene associated with the progression and prognosis of neuroblastoma^18^. A transcriptome analysis performed by our group in primary hepatocytes during the process of mouse liver regeneration revealed that the expression of *Mycn* was low in normal hepatocytes, but increased along with hepatocyte proliferation after partial hepatectomy^19^. We also reported that *MYCN* expression was seen in epithelial cell adhesion molecule (EpCAM)^+^ liver CSC-like cells and was positively correlated with the recurrence of HCC^20^. However, the mechanism underlying the overexpression of *MYCN* during chronic liver injury and hepatic tumorigenesis is still unclear.

Acyclic retinoid (ACR) is a synthetic vitamin A-like compound capable of preventing the recurrence of HCC in patients after curative removal of the primary tumors^21^. Recently, we identified that the MYCN high expression (MYCN^high^) liver CSC-like cells are selectively depleted by ACR, suggesting MYCN as a therapeutic target for the prevention and treatment of HCC^20^. Further proteome analysis showed enrichment in MYCN^high^EpCAM^+^ CSC-like HCC cells for lipogenic enzymes such as stearoyl-Coenzyme A desaturase-1 (SCD1), an enzyme that creates double bonds at specific locations in long chain fatty acids^22^. Therefore, in this study, we examined the link between lipid desaturation and *MYCN* gene expression in HCC cells.

## Results

### Metabolome characterization of MYCN^high^ CSC-like HCC cells

**Fig. 1:**
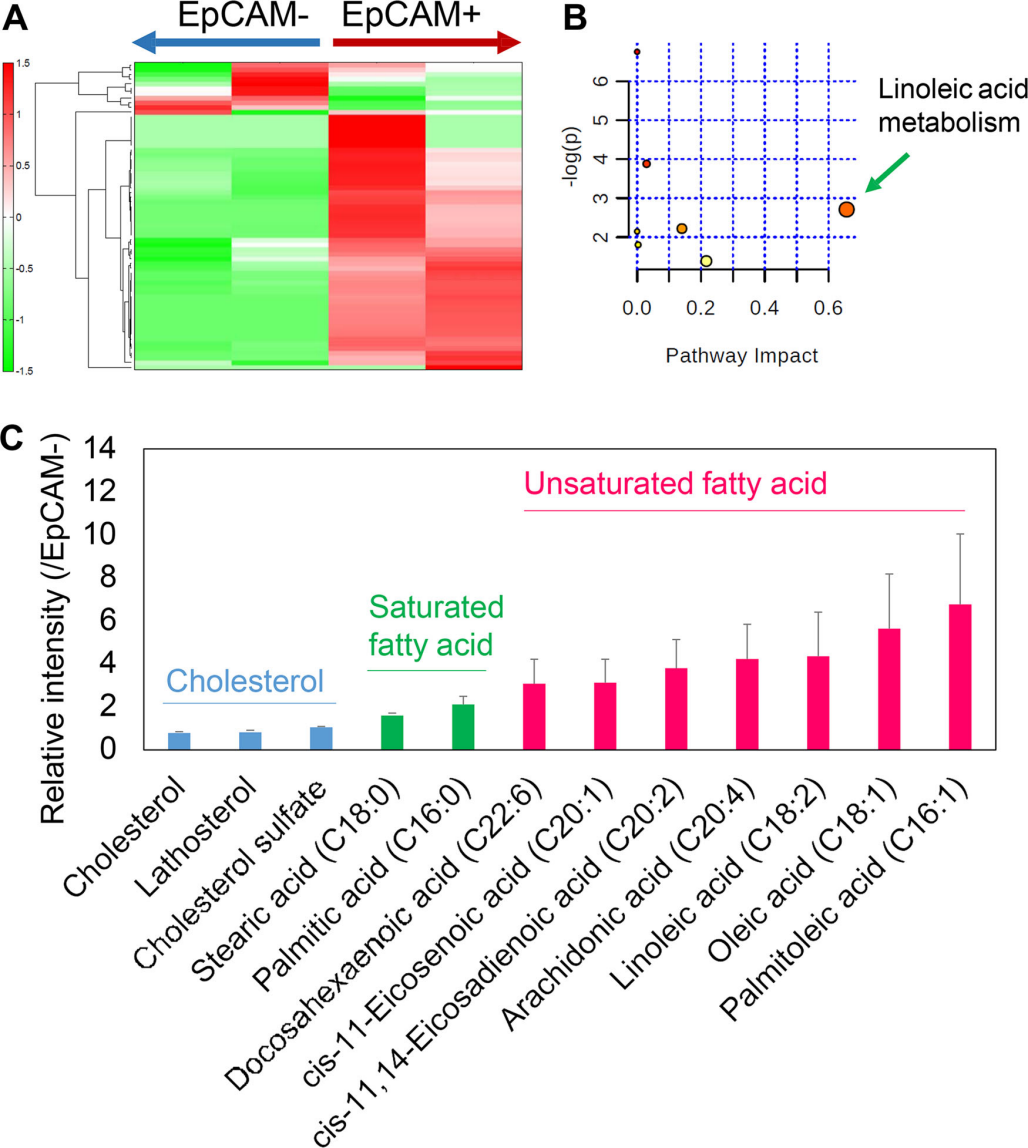
Metabolome characterization of EpCAM^+/−^ HCC cells. The sorted EpCAM^+/−^ JHH7 cells were used. **a** The clusters generated by hierarchical cluster analysis (HCA) were applied to the lipophilic metabolic profiles detected using a LC-TOFMS-based metabolomics technique. **b** The pathway impact analysis of differentially expressed metabolites with a fold change of more than 2 between EpCAM^+/−^ HCC cells using MetaboAnalyst 4.0. Metabolic pathways with values > 0.1 were considered to be perturbed. **c** Relative intensity of cholesterol, saturated fatty acids, and unsaturated fatty acids. The data are presented as the fold changes between EpCAM^+/−^ JHH7 cells.

To determine the metabolic characteristics of MYCN^high^ CSC-like HCC cells, metabolite analysis was performed on the EpCAM^+/−^ JHH7 cells sorted using fluorescence activated cell sorting (FACS). Peaks of a total of 65 lipophilic metabolites were detected using liquid chromatograph time-of-flight mass spectrometry (LC-TOFMS) (Table S1). Hierarchical cluster analysis (HCA) demonstrated a clear distinction in the abundance of lipophilic metabolites between EpCAM^+/−^ cells (Fig. 1a). The pathway impact analysis of the differentially expressed metabolites with a threshold of fold change of more than 2 using MetaboAnalyst showed that linoleic acid (LA; C18:2) metabolism was the most significantly perturbed pathway between EpCAM^+/−^ cells (Fig. 1b). Furthermore, in accordance with the proteome analysis^22^, the content of unsaturated fatty acids was strikingly increased in the EpCAM^+^ cells compared with that in the EpCAM^−^ cells. Palmitoleic acid (PA, C16:1; 6.8-fold) and oleic acid (OA, C18:1; 5.6-fold), which are the main monounsaturated fatty acids generated via SCD1^23^, were the two most dramatically enhanced lipophilic metabolites in EpCAM^+^ cells. In contrast, there were modest increases in the content of saturated fatty acids such as stearic acid (SA, C18:0; 1.6-fold) and palmitic acid (C16:0; 2.1-fold), and almost no changes in cholesterol (0.79-fold) and cholesterol sulfate (1.1-fold) in the EpCAM^+^ cells compared with those in the EpCAM^−^ cells (Fig. 1c).

### MYCN co-expression genes in HCC tumor tissues and cell lines

**Fig. 2:**
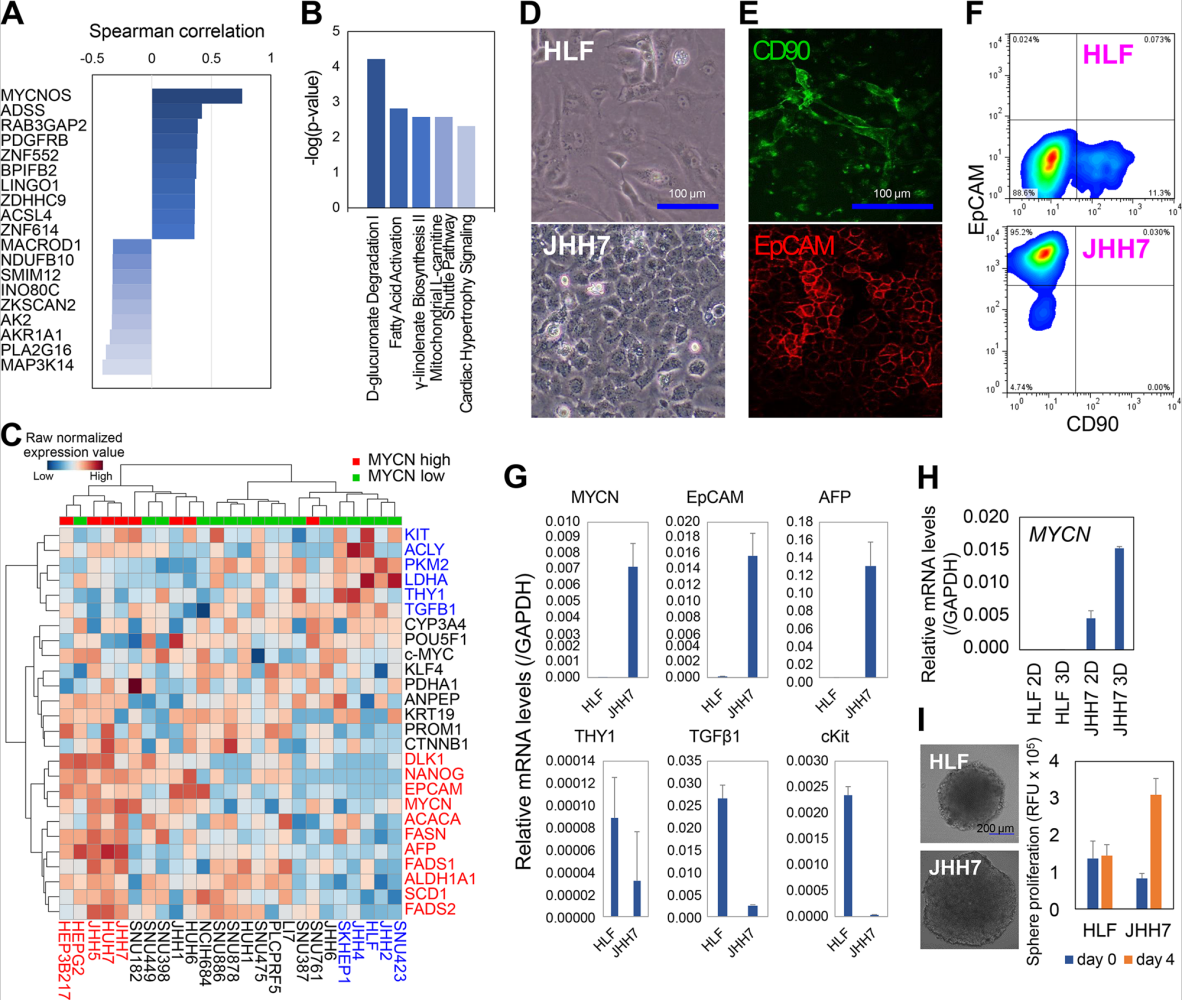
*MYCN* co-expression genes in HCC tumor tissues and cell lines. **a** Top *MYCN* co-expression genes selected from the liver hepatocellular carcinoma TCGA PanCancer RNA-seq data with a threshold of Spearman’s correlation coefficient of more than 0.3. **b** Top canonical pathways associated with *MYCN* co-expression genes in the IPA program. **c** Hierarchical clustering of *MYCN* and genes related with hepatic stem/progenitor markers and cancer metabolism and metastasis. MYCN high: HCC cells with the expression level of *MYCN* ≥ 1 RPKM; MYCN low: HCC cells with the expression level of *MYCN* *<* 1 RPKM. **d** Brightfield microscopic analysis, **e** immunofluorescence staining, and **f** flow cytometric analysis of CD90 or EpCAM expression in HLF and JHH7 cells. Scale bars, 100 μm. **g** PCR analysis of gene expression in HLF and JHH7 cell. **h** Gene expression of *MYCN* in JHH7 cells grown in monolayer (2D) and sphere cultures (3D) for 8 days. **i** Sphere proliferation of the HLF and JHH7 cells. RFU, relative fluorescent units. Scale bar, 200 μm. The data are presented as means (*n* = 3 replicates) ± SD.

Next, we undertook a clinical investigation of MYCN co-expression genes in human HCC tumor tissues. The genes were selected using The Cancer Genome Atlas (TCGA) database, which contains a RNA-seq dataset of 372 HCC tumor samples (PanCancer Atlas)^24^. With a threshold of Spearman’s correlation coefficient of more than 0.3, 109 genes were selected as MYCN co-expression genes and then subjected to pathway analysis (Fig. 2a). Ingenuity Pathway Analysis (IPA) revealed that the top canonical pathways such as the fatty acid activation and γ-linolenate biosynthesis were correlated with MYCN in HCC (Fig. 2b). Then, we examined the correlation between the MYCN gene expression and well-established hepatic stem/progenitor markers^25^ and genes associated with cancer metabolism and metastasis^26^ by data mining in the Cancer Cell Line Encyclopedia (CCLE) database^27^. With a threshold expression level of MYCN at 1 read per kilobase of exon per million mapped reads (RPKM), 8 out of a total of 25 HCC cell lines were selected as MYCN^high^ cells (≥1 RPKM), while the other 17 HCC cell lines were MYCN low expression (MYCN^low^) cells (<1 RPKM). Hierarchical clustering demonstrated enrichment in MYCN^high^ HCC cells for genes related with liver CSC markers (*EpCAM* and *AFP*), stemness regulation (*DLK1, NANOG*, and *ALDH1A1*), and fatty acid metabolism (*ACACA*, *FASN*, *FADS1, SCD1*, and *FADS2*) (Fig. 2c). In contrast, MYCN^low^ HCC cells were characterized by the expression of liver CSC marker CD90 (*THY1*) and genes involved in glucose metabolism (*PKM2* and *LDHA*) and epithelial-to-mesenchymal transition (*c-KIT* and *TGFβ1*) (Fig. 2c).

For further functional analysis, HLF and JHH7 cells were used as MYCN^low^ and MYCN^high^ HCC cells, respectively. Morphological analysis showed that the HLF cells had features of mesenchymal-like cells, while the JHH7 cells had those of epithelial cells (Fig. 2d). Immunofluorescence staining (Fig. 2e) and flow cytometric analysis (Fig. 2f) revealed that the HLF cells were negative for EpCAM and positive for CD90, while in contrast JHH7 cells were positive for EpCAM and negative for CD90. PCR analysis confirmed that the gene expression levels of *MYCN*, *EpCAM*, and *AFP* were dramatically higher, while the expression levels of *THY1*, *TGFβ1*, and *c-KIT* were lower in the JHH7 cells than that in the HLF cells (Fig. 2g). Then, we examined the *MYCN* expression in a three-dimensional (3D) multicellular tumor spheroid system, which could mimic in vivo 3D tumor growth and promote stemness maintenance^28^. 3D culture increased the *MYCN* gene expression in JHH7 cells, but not in HLF cells (Fig. 2h). Furthermore, the JHH7 cells showed greater sphere growth ability than the HLF cells (Fig. 2i).

### Loss of function analysis of SCD1 in MYCN^high^ HCC cells

**Fig. 3:**
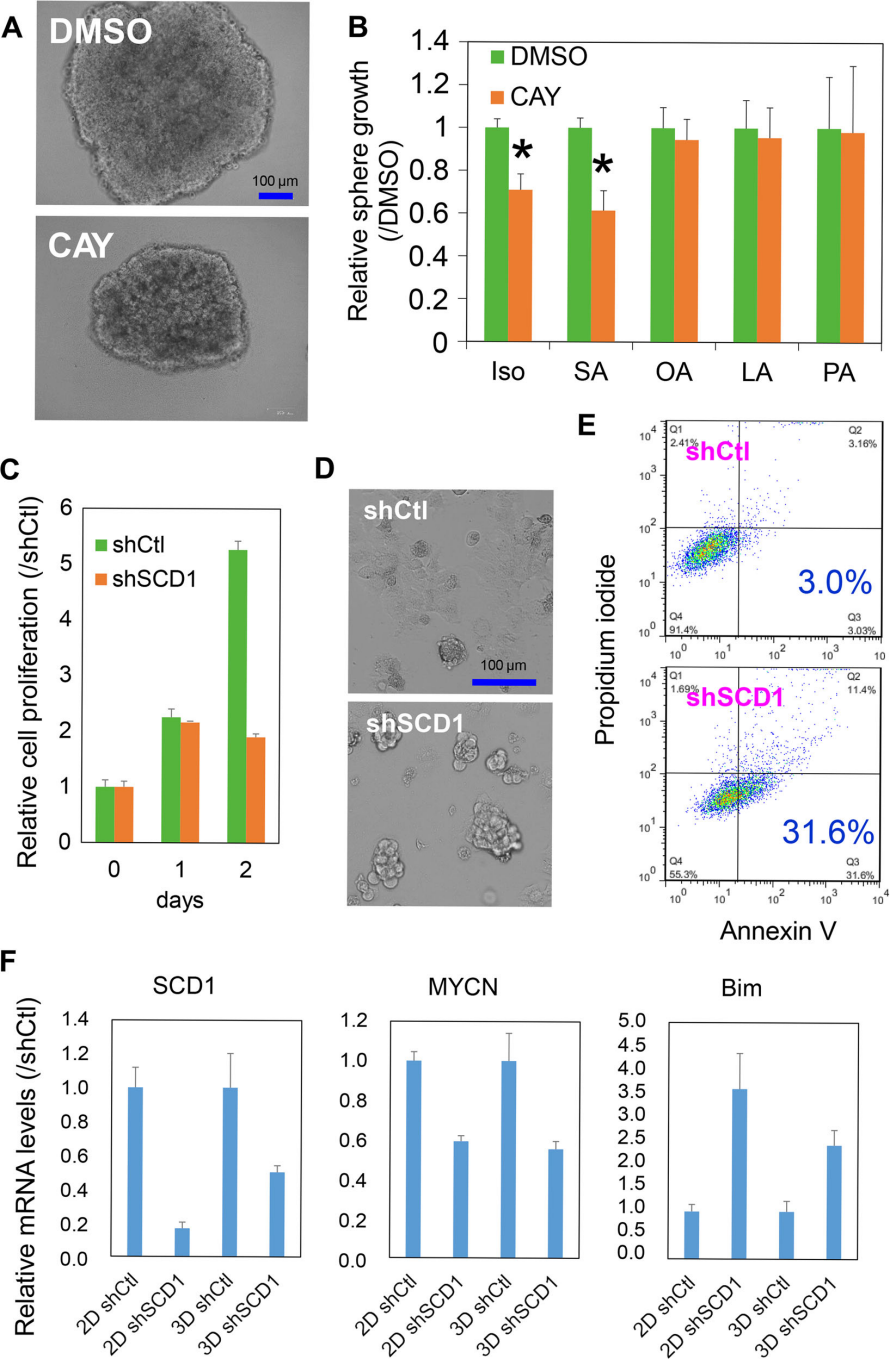
Loss of function analysis of SCD1 in MYCN^high^ HCC cells. **a** Representative microscopic images of JHH7 cells grown in sphere cultures. The spheres were treated with DMSO or a chemical inhibitor of SCD1, CAY10566 (CAY), at 10 μM for 4 days. Scale bar, 100 μm. **b** Sphere proliferation of JHH7 cells treated with DMSO or 10 μM CAY in the presence or absence of 100 μM fatty acids (stearic acid, SA; oleic acid, OA; linoleic acid, LA; palmitoleic acid, PA) for 7 days. Isopropanol (iso) was used as the primary solvent for the fatty acids. **c** Cell proliferation of control shRNA (shCtl) or shSCD1-transduced JHH7 cells grown in monolayer cultures. **d** Representative microscopic images of shCtl or shSCD1-transduced JHH7 cells grown in monolayer cultures for 3 days. Scale bar, 100 μm. **e** Apoptotic cell death of shCtl or shSCD1-transduced JHH7 cells grown in monolayer cultures for 3 days was detected by dual staining with Annexin V-FITC and propidium iodide (PI) followed by flow cytometric analysis. **f** Gene expression of *SCD1*, *MYCN*, and *BCL2L11* (Bim*)* in shCtl and shSCD1-transduced JHH7 cells in monolayer (2D) and sphere cultures (3D) for 7 days. The data are presented as means (*n* = 3 replicates) ± SD; *p *<* 0.05, Student’s *t*-test.

The above data suggested that *MYCN* was highly expressed in epithelial-like HCC cells with preferentially high tumorigenic potential and CSC characteristics, which was probably related with the acceleration of unsaturated fatty acid synthesis. To examine this hypothesis, we examined whether the inhibition of lipid desaturation using a chemical inhibitor (CAY10566) or shRNA against SCD1 might play a functional role in the proliferation of MYCN^high^ HCC cells in monolayer and sphere cultures. CAY10566 inhibited the spheroid proliferation in the JHH7 cell 3D culture system (Fig. 3a). To provide functional evidence supporting the lipid desaturation-mediated effects of CAY10566, we measured sphere proliferation with exogenously added lipid supplementation. The combined addition of monounsaturated fatty acids, OA and PA, and polyunsaturated fatty acid LA, but not the saturated fatty acid SA, restored the suppression of sphere proliferation by CAY10566 (Fig. 3b). Furthermore, we compared the effect of CAY10566 on the proliferation of MYCN^low^ HLF cells and MYCN^high^ JHH7 cells (Figure S1). Notably, a significantly greater growth suppressive effect of CAY10566 was observed in JHH7 cells than in HLF cells when grown as both monolayer and spheres. These data supported SCD1-mediated lipid desaturation as a major player in maintaining the sphere growth of MYCN^high^ HCC cells. Similarly, knockdown of *SCD1* using its shRNA inhibited the proliferation of JHH7 cells in monolayer culture (Fig. 3c) and there was an increased formation of apoptotic bodies (Fig. 3d). Furthermore, flow cytometric analysis demonstrated that the proportion of Annexin V positive apoptotic cells was increased among these cells (Fig. 3e). PCR analysis also revealed that the expression of pro-apoptotic gene Bcl-2-like protein 11 (*BCL2L11*; commonly called Bim) was increased following *SCD1* knockdown in JHH7 cells in both monolayer and sphere cultures (Fig. 3f). Moreover, knockdown of *SCD1* led to the decrease in *MYCN* gene expression in JHH7 cells, suggesting that SCD1-mediated signaling pathway might act as an upstream regulator of *MYCN* gene expression in HCC cells.

### RNA-seq based transcriptome analysis identifies molecular targets of SCD1 in MYCN^high^ HCC cells

**Fig. 4:**
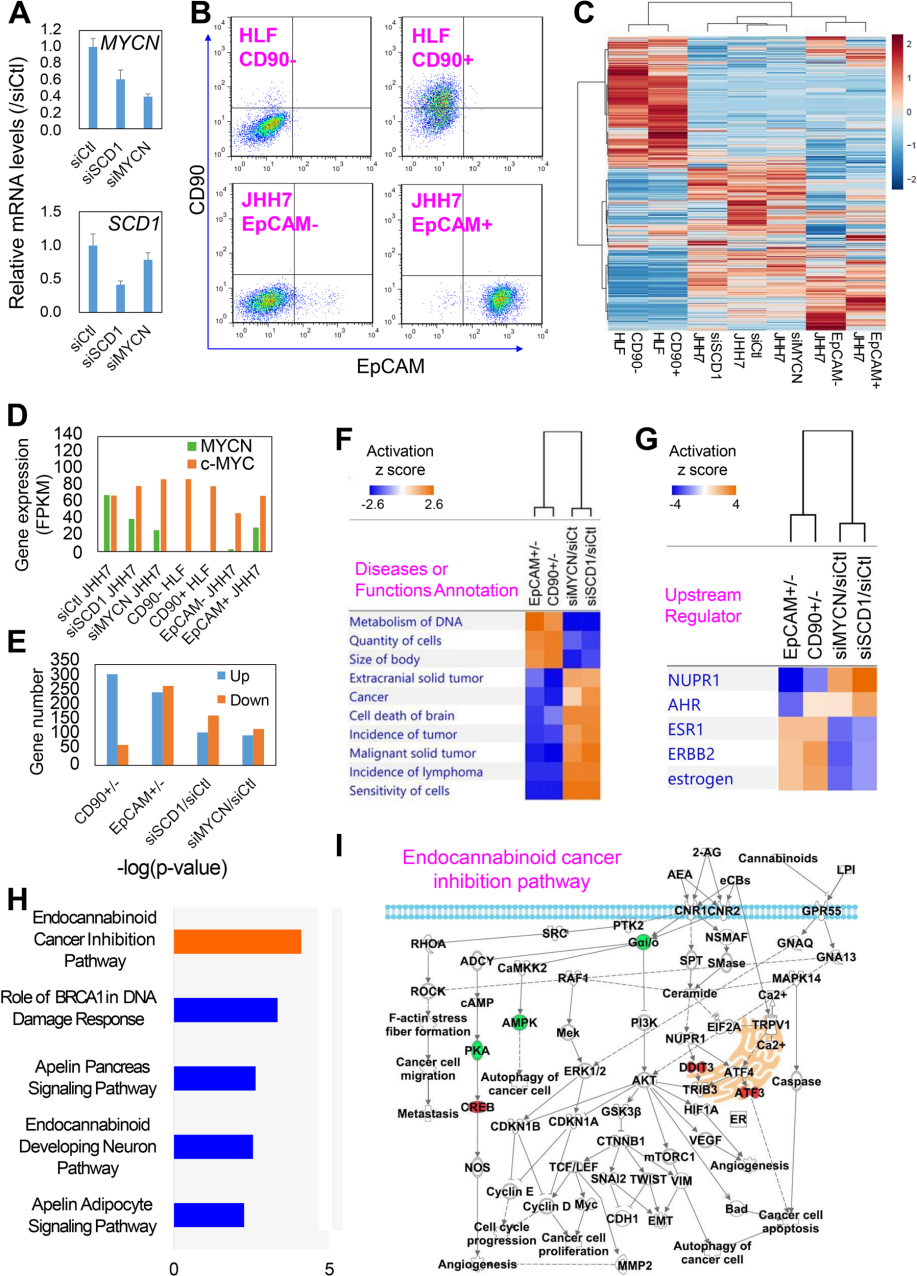
RNA-seq based transcriptome analysis identifies molecular targets of SCD1 in MYCN^high^ HCC cells. **a** *MYCN* (upper) and *SCD1* (lower) gene expression in JHH7 cells treated with 100 nM control siRNA (siCtl), siSCD1 or siMYCN for 24 h. **b** Double-staining flow cytometric analysis of CD90 and EpCAM in FACS-isolated CD90^+/−^ HLF cells (upper) and EpCAM^+/−^ JHH7 cells (lower). **c** Hierarchical clustering of RNA-seq based transcriptome profiles of CD90^+/−^ HLF cells, EpCAM^+/−^ JHH7 cells and JHH7 cells treated with 100 nM siCtl, siSCD1 or siMYCN for 24 h. **d** Expression of *MYCN* and *c-MYC* measured by RNA-seq analysis. FPKM, fragments per kilobase of exon model per million reads mapped. **e** Numbers of differentially expressed genes with a threshold of change of more than 4-fold. **f** Top disease or functional annotation and **g** upstream regulator associated with differentially expressed genes between EpCAM^+/−^ JHH7 cells, CD90^+/−^ HLF cells, siMYCN/siCtl-treated JHH7 cells, and siSCD1/siCtl-treated JHH7 cells performed in IPA platform. The pathways were ranked according to their activation z score, which can be used to predict either activated or inhibited state based on a statistically significant pattern match of up- and downregulation, respectively. **h** Top highly populated canonical pathways associated with a total of 154 genes (1) differentially expressed by siSCD1 transfection in JHH7 cells; (2) differentially expressed between EpCAM^+/−^ JHH7 cells and reversibly changed in compared with siSCD1 transfected JHH7 cells; (3) with no changes between CD90^+/−^ HLF cells and by siMYCN transfection in JHH7 cells. **i** A representative network under control of SCD1 in JHH7 cells called the endocannabinoid cancer inhibition pathway. Upregulated genes are indicated in red, downregulated genes indicated in green, and genes that were not annotated in this study, but are part of this network were indicated in white.

To explore the mechanism underlying the regulation of *MYCN* gene expression by SCD1, RNA-sequencing (RNA-seq) based transcriptome analysis was performed to identify the molecular targets of SCD1 in JHH7 cells with transient transfection of a control siRNA (siCtl) or siRNAs against *SCD1* (siSCD1) or *MYCN* (siMYCN) (Fig. 4a), and FACS-sorted CD90^+/−^ HLF cells and EpCAM^+/−^ JHH7 cells (Fig. 4b). Hierarchical clustering of the RNA-seq based transcriptome profiles with 16,735 genes demonstrated diverse expression profiles between HLF and JHH7 cells (Fig. 4c and Table S2). In accordance with the above PCR analysis, the *MYCN* gene expression was not detected in CD90^+/−^ HLF cells, while it was highly expressed in the EpCAM^+^ JHH7 cells as compared with that in the EpCAM^−^ cells and was decreased following knockdown of *SCD1* or *MYCN* in JHH7 cells (Fig. 4d). Furthermore, *MYCN* gene expression was not correlated with that of *c-MYC*, which is a major member of the MYC family contributing to the genesis of human cancers^16^ (Fig. 4d). With a threshold fold change of more than 4, differentially expressed genes between CD90^+/−^ HLF cells (383 genes), EpCAM^+/−^ JHH7 cells (524 genes), and siSCD1/siCtl (284 genes) and siMYCN/siCtl (228 genes) JHH7 cells were selected for pathway analysis to gain greater insights into the biological function (Fig. 4e). Top diseases or functional annotation (Fig. 4f) and upstream regulator analysis (Fig. 4g) using IPA platform revealed that EpCAM^+^ JHH7 cells and CD90^+^ HLF cells shared similar activation patterns, such as activation of DNA metabolism and nuclear protein 1 (Nupr1)-regulated signaling pathways, and inhibition in cell death program and estrogen-related signaling pathways. This data was in agreement with the previous reports that the stemness could be considered as a state acquired but not defined by a distinct cell type or a cell biomarker^29^. Notably, the CSC-related signaling pathways were reversibly affected by knockdown of either *SCD1* or *MYCN*, suggesting that SCD1 and/or MYCN might serve as potential CSC therapeutic targets. To identify the upstream regulators of *MYCN* gene expression under the control of SCD1, a total of 152 genes were further selected with a threshold fold change of more than 2 according to the following strategy: (1) differentially expressed by *siSCD1* transfection in JHH7 cells; (2) differentially expressed between EpCAM^+/−^ JHH7 cells and reversibly changed in comparison to the siSCD1 transfected JHH7 cells; (3) no changes between the CD90^+/−^ HLF cells and by siMYCN transfection in JHH7 cells. Canonical pathways analysis of these genes revealed that endocannabinoid signaling pathways were under the control of SCD1 in HCC cells (Fig. 4h). The most highly populated network was the endocannabinoid cancer inhibition pathway, which contains genes that play critical roles in controlling endoplasmic reticulum (ER) stress such as cyclic AMP response element-binding protein (*CREB*), DNA damage inducible transcript 3 (*DDIT3*), and cyclic AMP-dependent transcription factor (*ATF3*) (Fig. 4i). These data suggested that the *SCD1* knockdown-induced ER stress might play a functional role in regulation of *MYCN* gene expression in the HCC cells.

### ER stress regulates MYCN gene expression in MYCN^high^ HCC cells

**Fig. 5:**
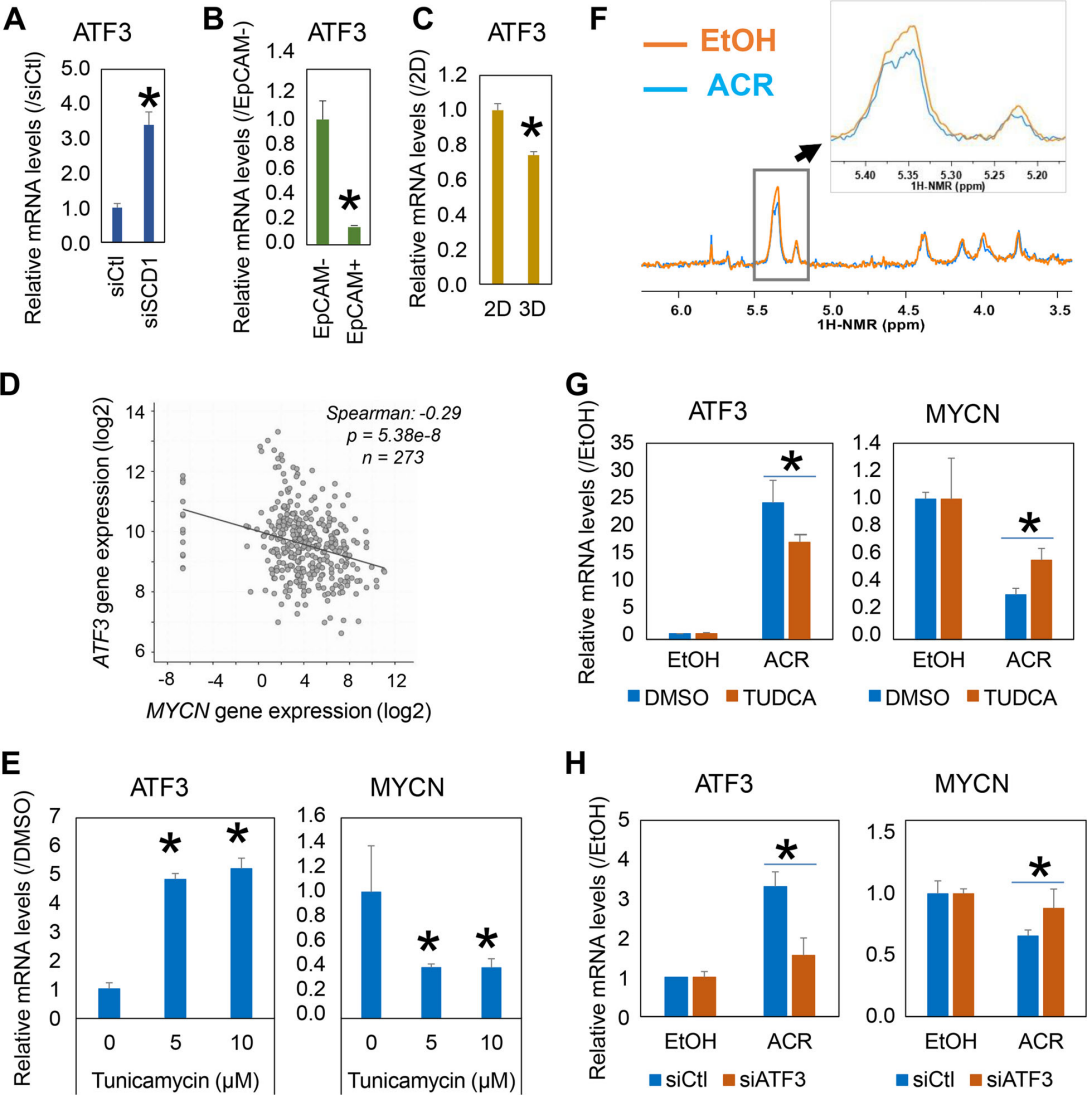
ER stress regulates *MYCN* gene expression in MYCN^high^ HCC cells. **a** *ATF3* gene expression in JHH7 cells treated with 100 nM control siRNA (siCtl) or siSCD1 for 24 h. **b** *ATF3* gene expression in FACS-isolated EpCAM^+^ and EpCAM^−^ JHH7 cells. **c** *ATF3* gene expression in JHH7 cells grown as monolayers (2D) and spheres (3D). **d** Correlation between *MYCN* and *ATF3* gene expression levels in HCC tissues of 372 patients obtained from the liver hepatocellular carcinoma TCGA PanCancer RNA-seq database. **e** Effect of ER stress inducer tunicamycin on *ATF3* (left) and *MYCN* (right) gene expression in JHH7 cells. The cells were treated with tunicamycin at indicated concentrations for 4 h. **f** Expansion of the ^1^H NMR spectrum of JHH7 cells treated with EtOH or 15 μM ACR for 4 h. The double bond region of unsaturated fatty acids from 5.20 to 5.45 ppm were highlighted. **g** Effect of ER stress inhibitor tauroursodeoxycholic acid (TUDCA) on ACR-mediated gene expression of *ATF3* (left) and *MYCN* (right) in JHH7 cells. The cells were treated with EtOH or 15 μM ACR in the absence or presence of 1 mM TUDCA for 4 h. **h** Effect of siATF3 on ACR-mediated gene expression of *ATF3* (left) and *MYCN* (right) in JHH7 cells. The cells were transfected with 100 nM siCtl or siATF3 for 24 h and then treated with EtOH or 15 μM ACR for 4 h. The data are presented as means (*n* = 3 replicates) ± SD; *p *<* 0.05, Student’s *t*-test.

The gene expression of *ATF3* was examined using PCR. Knockdown of *SCD1* induced the significant expression of *ATF3* in JHH7 cells (Fig. 5a). In contrast, the gene expression of *ATF3* was decreased in the EpCAM^+^ CSC-like JHH7 cells than that in the EpCAM^−^ JHH7 cells (Fig. 5b). The gene expression of *ATF3* was also decreased in JHH7 cells grown in sphere cultures than that in monolayer cultures (Fig. 5c). Data mining in the TCGA database revealed a significant negative correlation between the gene expression of *MYCN* and *ATF3* in human HCC tissues (Fig. 5d). Since *ATF3* is an ER stress-inducible transcriptional repressor^30^, we then examined whether ER stress might functionally regulate *MYCN* gene expression. Tunicamycin, a chemical inducer of ER stress, strongly stimulated the gene expression of *ATF3* and inhibited the gene expression of *MYCN* in the JHH7 cells (Fig. 5e). Finally, we examined whether ER stress is associated with the suppression of *MYCN* gene expression by ACR. Lipid profiling using ^1^H nuclear magnetic resonance (NMR) spectroscopy revealed that the treatment with ACR for 4 h dramatically reduced the content of unsaturated fatty acids in JHH7 cells (Fig. 5f). To further explore the molecular basis of the suppressive effect of ACR on lipid desaturation, the effect of ACR on gene expression of lipid desaturases was examined in JHH7 cells (Figure S2). Treatment with ACR for 4 h significantly suppressed the gene expression of *SCD1* as well as fatty acid desaturase-1 (*FADS1*) in JHH7 cells. ACR significantly enhanced the gene expression of *ATF3* in JHH7 cells, while co-addition of tauroursodeoxycholic acid (TUDCA), a chemical inhibitor of ER stress (Fig. 5g), as well as knockdown of *ATF3* using siRNA (Fig. 5h) significantly diminished the effects of ACR on *ATF3* and *MYCN* gene expression in JHH7 cells. These data suggested ACR suppressed *MYCN* gene expression in JHH7 cells, at least in part through ER stress induced ATF3 signaling pathways.

## Discussion

We have previously reported that (1) inhibition of biosynthesis of unsaturated fatty acids, but not glucose metabolism plays a crucial role in the prevention of hepatocellular carcinogen diethylnitrosamine (DEN)-induced hepatic tumorigenesis by ACR in the obesity mice model^31^; (2) ACR can inhibit *MYCN* gene expression and selectively kill MYCN^high^ EpCAM^+^ CSC-like HCCs, thus, providing a molecular basis by which ACR prevents HCC recurrence^20^; (3) proteome analysis has shown that MYCN^high^ EpCAM^+^ CSC-like HCC cells are enriched in enzymes involved in lipid desaturation such as SCD1, FADS1 and FADS2^22^. It should be noted that it is the upregulation of *MYCN* mRNA expression, but not its amplification status that is correlated with the prognosis of HCC^20^. Therefore, it seems that *MYCN* gene expression might represent a dynamic stem state of the liver CSC-like cells and an understanding of how *MYCN* transcription is activated would provide new insights on liver tumorigenesis. In this study, we further provided evidence that (1) the content of unsaturated fatty acids was increased in MYCN^high^ EpCAM^+^ CSC-like HCC cells; (2) inhibition of lipid desaturation using either the chemical inhibitor or siRNA/shRNA against SCD1 suppressed cell proliferation as well as *MYCN* gene expression in the MYCN^high^ HCC cells grown as both monolayer and spheres. These data bridge the gap in the knowledge about the link between lipid desaturation and *MYCN* gene expression in HCC cells.

It is known that MYC family oncogenes activate the expression of the enzymes involved in fatty acid biosynthesis and promote lipid metabolic reprogramming in support of reprogrammed cell growth and survival in tumorigenesis^32^. Blocking unsaturated fatty acid synthesis with chemical inhibitors had been shown to be toxic to Myc-overexpressing cells^10^. In contrast, our study showed a regulatory role of SCD1 on the *MYCN* gene expression in HCC cells, suggesting that, in addition to its potential as the major source of energy and building blocks of membranes for cell proliferation, the lipid-rich microenvironment might also contribute to tumorigenesis via mediating oncogenic signaling. Obesity might cause chronic inflammation via promoting the production of tumor-promoting cytokines such as IL-6 and activation of oncogenes such as *STAT3*, which has a critical role in regulating cell fate, inflammation, and immunity^33^. A new study has demonstrated that the fatty acids could directly activate STAT3 by enhancing its palmitoylation^34^. In addition, there is increasing focus on the emerging roles of lipid metabolism, especially SCD1-mediated lipid desaturation, in the generation and maintenance of CSCs, through the activation of oncogenic signaling pathways such as NF-κB, Wnt/β-catenin and Hippo/YAP signaling^35^. For example, monounsaturated fatty acids such as OA might support mRNA stabilization of the Wnt receptor LRP5/6 and contribute to the activation of Wnt/β-catenin signaling, which is critical for maintaining stem cell pluripotency^36^. Therefore, targeting lipid desaturation should be a promising therapeutic strategy for HCC treatment via the inhibition of energy production and membrane building blocks, as well as for HCC prevention through the suppression of oncogenic signaling and elimination of CSCs.

We further showed through transcriptome analysis that the ER stress related signaling networks such as endocannabinoid cancer inhibition pathway were under the control of SCD1 in MYCN^high^ HCC cells. Furthermore, the chemical inducer of ER stress inhibited *MYCN* gene expression, while the chemical inhibitor of ER stress partially rescued the suppression of *MYCN* gene expression by ACR in MYCN^high^ HCC cells. Lipid messengers, such as sphingolipids, lysolipids, and endocannabinoids, play critical roles in multiple cellular functions from cell proliferation to apoptosis and are emerging as promising therapeutic targets in cancer^37^. Endocannabinoids are endogenous lipid-based retrograde neurotransmitters and their G-protein-coupled receptors CB1 and CB2 are expressed throughout the nervous system. CB1 activation causes the generation of the lipid second messenger ceramide, which plays central roles in cannabinoid induced apoptosis, partly through triggering of ER stress pathways^38^. The *MYCN* oncogene is notably amplified in peripheral and central nervous system tumors especially neuroblastoma^18^. In addition, retinoic acids have been shown to transcriptionally induce *CB1* expression in hepatocytes via retinoic acid receptor γ-dependent pathways^39^. Therefore, it is possible that ACR regulates *MYCN* gene expression through endocannabinoid system-mediated ER stress signaling pathways.

It has been reported that decrease in membrane phospholipid unsaturation and increase in cellular saturated fatty acids induced ER stress-dependent cell death^40,41^. Indeed, activation of ER stress signaling is instrumental in the development of NASH-related HCC by triggering chronic liver damage and inflammation^7^. In contrast, tissue stem cells, as well as CSCs, are featured with their adaptive plasticity to allow them to survive when exposed to a wide range of stresses including ER stress^42^. Reduction in ER stress enables maintenance of functional hematopoietic stem cells^43^, while induction of ER stress causes loss of intestinal epithelial stemness^44^. Importantly, rescue of ER stress in CSC induces differentiation and sensitizes them to chemotherapy in colon cancer^45^ and HCC^46^. In agreement with this, we found that the expression of ER stress-inducible gene *ATF3* was downregulated in MYCN^high^ EpCAM^+^ CSC-like HCC cells and CSC-rich spheroids, which was upregulated by ACR treatment or lipid desaturation inhibition. Therefore, escaping from the ER stress-mediated programmed cell death in obesity-caused inflammatory microenvironment should play a critical role in the maintenance of liver CSCs and hepatic tumorigenesis and serve as a promising therapeutic target in the prevention of HCC.

In summary, multi-omics analyses revealed that lipid desaturation-mediated ER stress signaling reversibly regulates the *MYCN* gene expression in HCC cells and might be a promising therapeutic target for the treatment and prevention of HCC.

## Materials and methods

### Cell culture

The HCC cell line HLF (JCRB0405) was obtained from the Japanese Collection of Research Bioresources Cell Bank^47^. The HCC cell line JHH7 was kindly provided by Prof. T. Matsuura of the Jikei University School of Medicine, Tokyo, Japan^48^. The cells were maintained at 37 °C and 5% CO_2_, in Dulbecco’s modified Eagle medium (DMEM, Wako Industries, Osaka, Japan) containing 10% fetal bovine serum (FBS; Mediatech, Herndon, VA, USA), 100 U/mL penicillin/streptomycin, and 2 mM l-glutamine (Mediatech).

### Chemicals

A SCD1 inhibitor, CAY10566 (ab144421), was purchased from Abcam (Cambridge, MA, USA). Tunicamycin (11445) and TUDCA (20277) were purchased from Cayman Chemical (Ann Arbor, MI, USA). Fatty acids SA (S4751), OA (O1008), PA (P0500), and LA (L1376) were obtained from Sigma (Louis, MO, USA). ACR was supplied by Kowa Co. Ltd (Tokyo, Japan). Dimethyl sulfoxide (DMSO, Sigma) was used as the primary solvent for CAY10566, tunicamycin, and TUDCA. Isopropanol (Wako Industries) was used as the primary solvent for fatty acids. Ethanol (EtOH, Wako Industries) was used as the primary solvent for ACR.

### FACS sorting

Flow cytometric analysis was performed as previously described^20^. The cells were incubated with Alexa Fluor 647-conjugated mouse anti-EpCAM (1:20; 324212; BioLegend, San Diego, CA, USA) and/or FITC-conjugated mouse anti-CD90 (328108; BioLegend) at 4 °C for 30 min. The labeled cells were analyzed and sorted on a BD FACSAria (BD Biosciences, San Diego, CA, USA). The data were analyzed in FlowJo software (Tree Star, Inc., Ashland, OR, USA).

### LC-TOFMS metabolome analysis

The cells were washed with 5% (w/w) mannitol solution twice and harvested in EtOH containing 10 μM internal standards (Human Metabolome Technologies Inc., Tsuruoka, Japan) with scrapers. The cell pellet was diluted in 1000 μL Milli-Q water and supernatant was collected by centrifugation (4400 × *g*, 4 °C, 5 min). The supernatant was dried and resuspended in 200 μL of isopropanol/Milli-Q water solution (1:1) for LC-TOFMS analysis using an Agilent 1200 series RRLC system SL equipped with an Agilent LC/MSD TOF system (Agilent Technologies, Palo Alto, CA, USA)^31^.

### 3D spheroid cultures

3D spheroid cultures were performed on non-adherent 96-well round-bottomed Sumilon PrimeSurface™ plates (MS-9096U, Sumitomo Bakelite, Tokyo, Japan) as previously described^28^. The spheroids were grown for 4 days, then 50 µL of media was replaced with 50 µL fresh serum-free media containing 10 µM CAY10566, and/or 100 µM fatty acids. The spheroids were further cultured for 7 days and photos were taken using an optical microscope (DS-Fi1, NIKON, Tokyo, Japan).

### Spheroid proliferation assay

Spheroid proliferation was measured using the CellTiter-Fluor™ Cell Viability Assay (Promega Corporation, Madison, WI, USA). The fluorescence was measured using a plate reader (λ excitation/emission: 390 nm/505 nm) (ARVO MX, Perkin Elmer Inc., Waltham, MA, USA).

### In vitro RNA interference

A pool of 3 target-specific siRNAs targeting human *SCD1* (sc-36464; siSCD1), human *MYCN* (sc-36003; siMYCN), human *ATF3* (sc-44283; siATF3) and a control siRNA (sc-37007; siCtl) were purchased from Santa Cruz Biotechnology (Santa Cruz, CA, USA). The cells were plated in 24-well plates for 24 h, and then transfected with 100 nM siRNAs using lipofectamine 2000 transfection reagent (Life Technologies, Gaithersburg, MD, USA). On the following day, cells were collected for RNA isolation or treated with chemicals for further analysis.

### Transduction of shRNA lentiviral particles

*SCD1* (sc‑36464‑V) and control (sc‑108080) short hairpin RNA (shRNA) lentiviral particles were obtained from Santa Cruz Biotechnology. The cells were transduced with lentiviral vectors expressing the shRNAs at ~0.5 multiplicity of infection (MOI) using 5 μg/mL Polybrene (Santa Cruz Biotechnology) and then selected with 2 μg/mL puromycin‑containing culture medium.

### Immunofluorescence analysis

The cells were seeded in 96-well plates at 10^5^ cells/mL. After 24 h, the cells were stained with Alexa Fluor 647-conjugated mouse anti-EpCAM (1:20; 324212; BioLegend) and/or FITC-conjugated mouse anti-CD90 (1:20; 328108; BioLegend) at room temperature for 30 min. Images were captured using an ImageXpress Micro Confocal High‑Content Imaging System (Molecular Devices, Sunnyvale, CA, USA).

### RNA isolation and real-time (RT)-PCR

Total RNA was isolated using a FastGene RNA Basic Kit (FG-80250, NIPPON Genetics, Tokyo, Japan) and quantified using a NanoDrop spectrophotometer (NanoDrop products, Wilmington, DE, USA). cDNA was synthesized using a PrimeScript RT Master Mix Kit (TaKaRa Bio, Otsu, Japan). Primer sequences were as follows: glyceraldehyde 3-phosphate dehydrogenase (*GAPDH*) forward (5′ CAATGACCCCTTCATTGACC 3′) and reverse (5′ GACAAGCTTCCCGTTCTCAG 3′), *SCD1* forward (5′ GTACCGCTGGCACATCAACTT 3′) and reverse (5′ TTGGAGACTTTCTTCCGGTCAT 3′), *FADS1* forward (5′ CTACCCCGCGCTACTTCAC 3′) and reverse (5′ CGGTCGATCACTAGCCACC 3′), *MYCN* forward (5′ GGCAGTAGGACCACCAGTGT 3′) and reverse (5′ AACCGTCACCAACGTTTAGC 3′), *BCL2L11* forward (5′ GGCCCCTACCTCCCTACA 3′) and reverse (5′ GGGGTTTGTGTTGATTTGTCA 3′), and *ATF3* forward (5′ TTTGCTAACCTGACGCCCTT 3′) and reverse (5′ TGACTGATTCCAGCGCAGAG 3′). PCR reactions were performed using a Roche LightCycler 96 Real-Time PCR System (Roche Diagnostic Co., Ltd., Tokyo, Japan) and the SYBR Premix ExTaq II (TaKaRa Bio).

### Data mining

RNA-seq transcriptome profiles of 372 HCC tumor tissues (PanCancer Atlas) were obtained from the TCGA database using cBioPortal software (www.cbioportal.org/)^49^. RNA-seq transcriptome profiles of 25 HCC cell lines were obtained from the CCLE database (https://portals.broadinstitute.org/ccle)^27^.

### Cell viability assay

Cell viability was measured using a Cell Counting Kit-8 (Dojindo Molecular Technologies, Tokyo, Japan). The absorbance was measured using a plate reader (ARVO MX, Perkin Elmer Inc.) at 450 nm.

### Annexin V staining assay

Apoptotic cells were detected using the FITC Annexin V Apoptosis Detection Kit with Propidium iodide (PI) (640914, BioLegend). Briefly, the cells were washed thoroughly with cell staining buffer (420201, BioLegend) and resuspended in Annexin binding buffer. The cells were then labeled with Annexin V-FITC and PI at room temperature for 15 min in the dark. The fluorescence of stained cells was measured using a BD LSR flow cytometer and CellQuest Pro software (BD Biosciences). The data were further analyzed in FlowJo software (Tree Star, Inc.).

### RNA-seq based transcriptome analysis

RNA-Seq libraries were prepared using TruSeq Stranded mRNA Library Prep Kit (Illumina, San Diego, CA, USA), followed by paired-end sequencing on a NovaSeq 6000 instrument (Illumina). STAR (2.5.1b) was used to align reads in fastq files to the UCSC human hg38 reference genome. Stringtie (1.3.5) was used to assemble the transcriptome based on the hg38 reference annotation with Gencode.v30. The quantification of relative abundance of each transcript was reported as fragments per kilobase of exon model per million reads mapped (FPKM). Data downloads are available (Table S2).

### Knowledge-based pathway analysis

For the metabolome analysis, the pathway impact was calculated as the sum of the importance measures of the matched metabolites normalized by the sum of the importance measures of all metabolites in each pathway using MetaboAnalyst 4.0^50^. For the transcriptome analysis, the knowledge-based functional analysis was performed using the IPA platform (Ingenuity Systems, Mountain View, CA, USA) as previously described^19^. The generated biological networks were ranked by score, which is the likelihood of a set of genes being found in the network owing to random chance, identified by a Fisher’s exact test. The generated diseases or functional annotations or upstream regulators were ranked by the activation z score, which can be used to find likely regulating molecules based on a statistically significant pattern match of up- and downregulation, and also to predict the activation state (either activated or inhibited) of a putative regulator. An absolute z score of more than 2 was considered as significant.

### Lipid profiling using ^1^H NMR spectroscopy

The JHH7 cells treated with EtOH or 15 μM ACR for 4 h were harvested and lipid was extracted using the chloroform/methanol/water extraction method as previously described^51^. The ^1^H NMR spectra were measured at 700 MHz on a Bruker AVANCE II 700 spectrometer (Bruker BioSpin GmbH, Rheinstetten, Germany). All NMR spectra were processed using the MestReNova program (Version 12.0.1, MestRec, Santiago de Compostela, Spain). Tetramethylsilane was used as the internal standard. The double bond regions of unsaturated fatty acids appear mainly from 5.20 to 5.45 ppm.

### Statistical analysis

Quantitative data are expressed as the mean ± SD of at least three replicates. The significance of differences between values was assessed using Student’s *t*-test. A *p*-value < 0.05 was considered significant.

## Supplementary information

Supplementary Table and Figure Legends

Fig S1

Fig S2

Table S1

Table S2
