# Supplementary material for: Lipid desaturation-associated endoplasmic reticulum stress regulates MYCN gene expression in hepatocellular carcinoma cells
Source: Cell Death Dis. 2020 Jan 27;11(1):66. doi: 10.1038/s41419-020-2257-y (PMC6985230; doi:10.1038/s41419-020-2257-y)
Supplement: Supplementary file 1 — Supplementary Table and Figure Legends [file 41419_2020_2257_MOESM1_ESM.docx]

**Supplementary Table and Figure Legends**

**Table S1: Quantification of lipophilic metabolites in sorted EpCAM+/- JHH7 cells using LC-TOFMS.**

**Table S2: Raw datasets of RNA-seq transcriptome analysis.**

**Figure S1. Growth suppressive effects of CAY10566 in MYCN^low^ and MYCN^high^ HCC cells.** (**A**) Representative microscopic images and (**B**) relative cell viability of HLF and JHH7 cells grown as monolayers, treated with DMSO or 10 μM CAY10566 for 48 h. (**C**) Representative microscopic images and (**D**) relative sphere proliferation of HLF and JHH7 cells grown as spheres, treated with DMSO or 10 μM CAY10566 for 3 days. Scale bars, 100 μm. The data are presented as means (n = 3 replicates) ± SD. * *p* < 0.05, Student’s *t*-test.

**Figure S2. Effect of ACR on gene expression of lipid desaturates *SCD1* and *FADS1* in MYCN^high^ HCC cells.** JHH7 cells were treated with EtOH or 15 μM ACR for 4 h. The data are presented as means (n = 3 replicates) ± SD. * *p* < 0.05, Student’s *t*-test.
