# Supplementary figures and images for: Lipid desaturation-associated endoplasmic reticulum stress regulates MYCN gene expression in hepatocellular carcinoma cells
Source: Cell Death Dis. 2020 Jan 27;11(1):66. doi: 10.1038/s41419-020-2257-y (PMC6985230; doi:10.1038/s41419-020-2257-y)

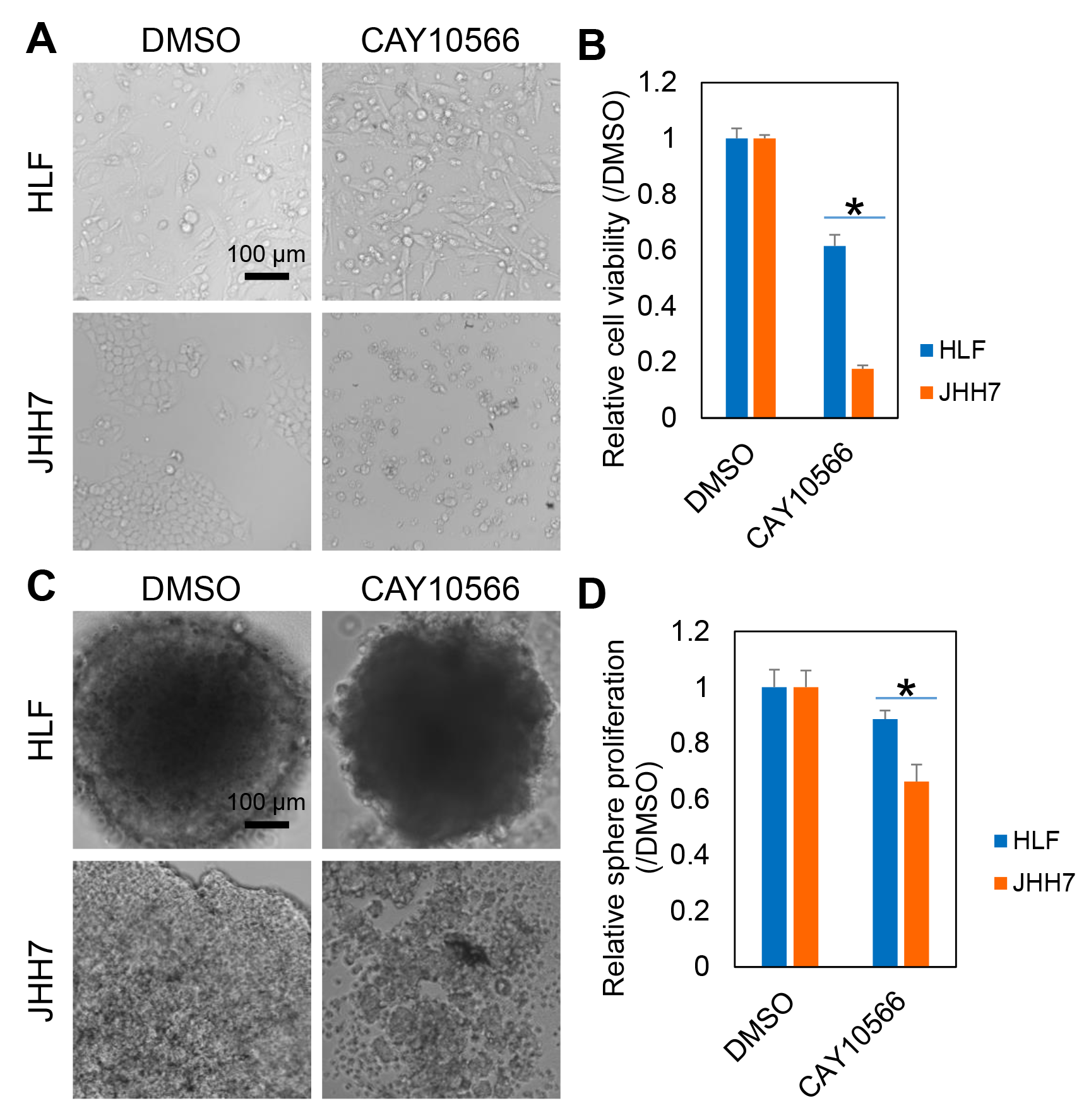

Supplement: Supplementary file 2 — Fig S1 [file 41419_2020_2257_MOESM2_ESM.tif]

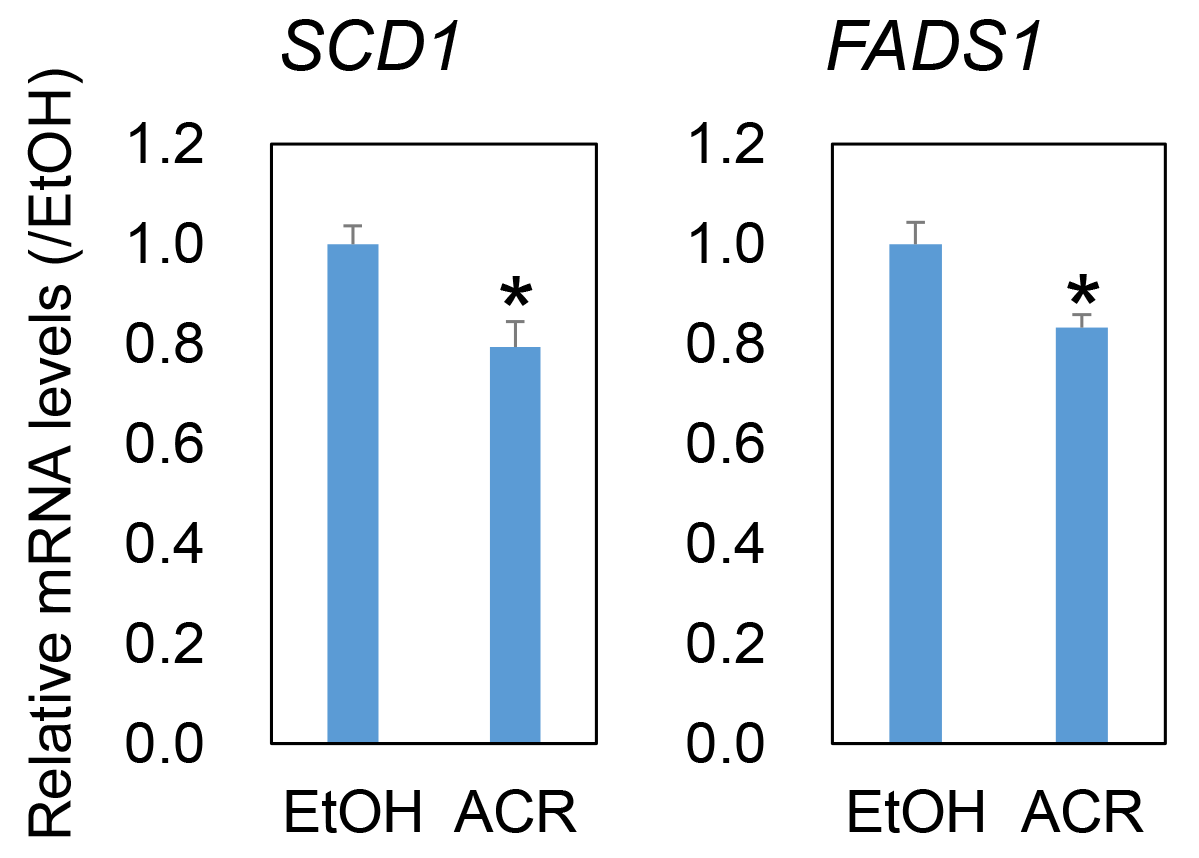

Supplement: Supplementary file 3 — Fig S2 [file 41419_2020_2257_MOESM3_ESM.tif]
